# Supplementary material for: Deficiency of intestinal Bmal1 prevents obesity induced by high-fat feeding
Source: Nat Commun. 2021 Sep 7;12:5323. doi: 10.1038/s41467-021-25674-5 (PMC8423749; doi:10.1038/s41467-021-25674-5)
Supplement: Supplementary file 1 — Supplementary Information File [file 41467_2021_25674_MOESM1_ESM.pdf]

Supplementary Information

Manuscript title:

Deficiency of intestinal *Bmal1* prevents obesity induced by high-fat feeding

Supplementary Table 1. Oligonucleotides used in this study.

	Forward (5'-3' sequence)	Reverse (5'-3' sequence)
**qPCR**		
Bmal1	CTCCAGGAGGCAAGAAGATTC	ATAGTCCAGTGGAAGGAATG
Npas2	AAGGATAGAGCAAAGAGAGCCT	CATTTTCCGAGTGTTACCAGGG
Rev-erbα	TTTTTCGCCGGAGCATCCAA	ATCTCGGCAAGCATCCGTTG
Rev-erbβ	GGAGTTCATGCTTGTGAAGGCTGT	CAGACACTTCTTAAAGCGGCACTG
Per1	GAAAGAAACCTCTGGCTGTTCCT	GCTGACGACGGATCTTTCTTG
Per2	CCACACTTGCCTCCGAAATA	ACTGCCTCTGGACTGGAAGA
Per3	CCGCCCCTACAGTCAGAAAG	GCCCCACGTGCTT AAATCCT
Cry1	CACTGGTTCCGAAAGGGACTC	CTGAAGCAAAAATCGCCACCT
Cry2	CACTGGTTCCGCAAAGGACTA	CCACGGGTCGAGGATGTAGA
Clock	AGAACTTGGCATTGAAGAGTCTC	GTCAGACCCAGAATCTTGGCT
E4bp4	CTTTCAGGACTACCAGACATCCAA	GATGCAACTTCCGGCTACCA
Dbp	ACATCTAGGGACACACCCAGTC	AAGTCTCATGGCCTGGAATG
Cd36	CCAAATGAAGATGAGCATAGGACAT	GTTGACCTGCAGTCGTTTTGC
Fabp2	GTGGAAAGTAGACCGGAACGA	CCATCCTGTGTGATTGTCAGTT
Mogat2	TGGGAGCGCAGGTTACAGA	CAGGTGGCATACAGGACAGA
Dgat1	TCCGTCCAGGGTGGTAGTG	TGAACAAAGAATCTTGCAGACGA
Dgat2	GGCTACGAACTATACAAGACGC	AGGATCAGCCAATTTATTGCTGG
Gpat3	GGCCTTCGGATTATCCCTGG	CTTGGGGGCTCCTTTCTGAA
Mttp	AGCTTTGTCACCGCTGTGC	TCCTGCTATGGTTTGTTGGAAGT
Apob	TTGGCAAACTGCATAGCATCC	TCAAATTGGGACTCTCCTTTAGC
Fasn	GGAGGTGGTGATAGCCGGTAT	TGGGTAATCCATAGAGCCCAG
Plin3	CCACAGGATGCTGAAAAGG	TGATGTCCCTGAACATGCTG
Acls5	AAGGCATTGGTGCTGATAGG	TCAGGTCTTCTGGGCTAGGA
Fabp2	GTGGAAAGTAGACCGGAACGA	CCATCCTGTGTGATTGTCAGTT
Atgl	CAACGCCACTCACATCTACGG	GGACACCTCAATAATGTTGGCAC
Hsl	CCAGCCTGAGGGCTTACTG	CTCCATTGACTGTGACATCTCG
Mgl	CGGACTTCCAAGTTTTTGTCAGA	GCAGCCACTAGGATGGAGATG
Pparα	AGGCCGTTGCCACTGTTCAG	AGCCCTCTTCATCCCCAAGC
Cpt1α	TGCTGTCCTCCTTGATGAAC	GCTTAAGCACGTGCACAATC
Cpt1β	GCACACCAGGCAGTAGCTTT	CAGGAGTTGATTCCAGACAGGTA
Acot1	ATACCCCCTGTGACTATCCTGA	CAAACACTCACTACCCAACTGT
Scd1	TTCTTGCGATACACTCTGGTGC	CGGGATTGAATGTTCTTGTCGT
Srebp1c	GATGTGCGAACTGGACACAG	CATAGGGGGCGTCAAACAG
Pgc1α	TATGGAGTGACATAGAGTGTGCT	GTCGCTACACCACTTCAATCC
Ucp1	AGGCTTCCAGTACCATTAGGT	CTGAGTGAGGCAAAGCTGATTT

Npc1l1	CGCCCTTCTTTCTACATGGGT	GAATCTGCGCTTACGAGGGAG
Abcg5	GTCCTGCTGAGGCGAGTAAC	CGCCCTTTAGCGTGTTGTTC
Abcg8	CTGTGGAATGGGACTGTACTTC	GTTGGACTGACCACTGTAGGT
Abca1	GTGGGCTCCTCCCTGTTTTT	TCTGAGAAACACTGTCCTCCTTTT
Acat2	CCCGTGGTCATCGTCTCAG	GGACAGGGCACCATTGAAGG
Hmgcr	ATGGCTGGGAGCATAGGCGG	CTGCATCCTGGCCACATGCG
Srebp2	GCAGCAACGGGACCATTCT	CCCCATGACTAAGTCCTTCAACT
Srb1	GGGAGCGTGGACCCTATGT	CGTTGTCATTGAAGGTGATGT
Sglt1	TGGGCTGGATATTTGTCCCGA	CAAACCGCTTCCGCAGATACT
Glut2	TCAGAAGACAAGATCACCGGA	GCTGGTGTGACTGTAAGTGGG
Glut5	CCAATATGGGTACAACGTAGCTG	GCGTCAAGGTGAAGGACTCAATA
Fatp1	CGCTTTCTGCGTATCGTCTG	GATGCACGGGATCGTGTCT
Fatp2	AACACATCGCGGAGTACCTG	CTCAGTCATGGGCACAAATG
Fatp4	GTGAAGGCAAAGGTGCGAC	CGGAAGGTCCAGTGGGTATC
Hmbs	CCGAGCCAAGGACCAGGATA	CCGAGCCAAGGACCAGGATA
BMAL1	AAATCGCTTTGAGGTGAC	CTTTCCGTTTGCGGTTGC
CD36	GGAGGACGCACCTGTTAGC	GTTGGTCTGAGGAGGAATGAAC
MOGAT2	TCCTTCACGCTGTTACTGCG	TCAAATAGGTCATTCTCCCCGAA
DGAT1	CAATCTGACCTACCGCGATCT	TCGATGATGCGTGAGTAGTCC
DGAT2	AGTGGCAATGCTATCATCATCGT	TCTTCTGGACCCATCGGCCCCAGGA
GPAT3	CTGCTGGTCGGCTTTGTCTT	TCCAGAGTGAGTAGGCGAGG
FASN	AAGGACCTGTCTAGGTTTGATGC	TGGCTTCATAGGTGACTTCCA
MTTP	ATTGTAAAGTGACCTACCAGGCT	ACCTCGCTATTTTGCATGAATCC
APOB	GGGAACAACGCTAGTCCCAC	CTCCTCTGGCTCAACAATCAG
PLIN3	TATGCCTCCACCAAGGAGAG	ATTCGCTGGCTGATGCAATCT
GAPDH	CATGAGAAGTATGACAACAGCCT	AGTCCTTCCACGATACCAAAGT
**EMSA**		
Dgat2	GGATCGTCCCGCACGTGCTCAAACTCC	GGAGTTTGAGCACGTGCGGGACGATCC
Dgat2 (mutant)	GGCTCATCACACGAGTGATCATACTCC	GGAGTATGATCACTCGTGTGATGAGCC
**CHIP**		
Dgat2-Ebox	AAGGCTGCGGCACTTGGGTA	AGAATACGGGCAAGGGAG
**siRNA**		
siBmal1	GCUCUUUCUUCUGUAGAAUTT	AUUCUACAGAAGAAAGAGCTT
siBMAL1	CCGAGGGAAGAUACUCUUUTT	AAAGAGUAUCUUCCCUCGGTT
siControl	UUCUCCGAACGUGUCACGUTT	ACGUGACACGUUCGGAGAATT

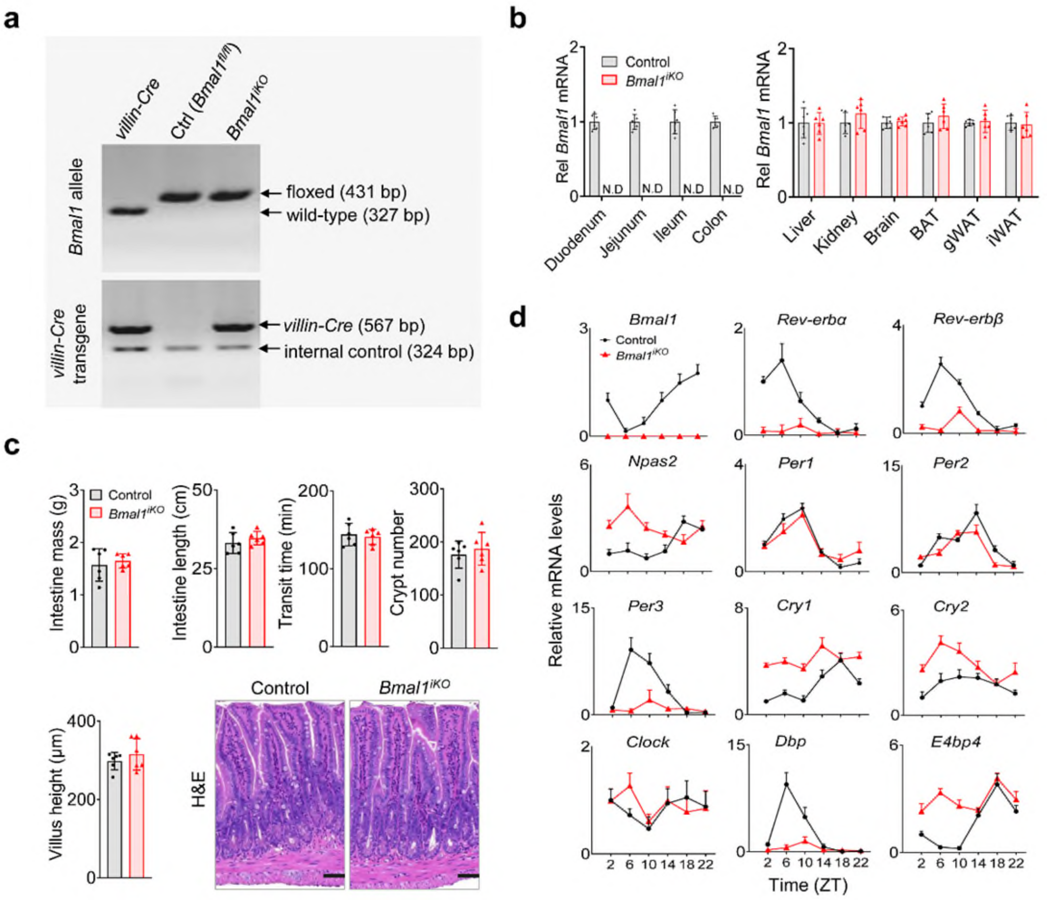

Supplementary Figure 1. (**a**) PCR-based genotyping of *Bmal1*^{iKO} mice. *Bmal1*^{iKO} mice had both floxed *Bmal1* allele and *villin-Cre*. (**b**) mRNA expression of *Bmal1* in duodenum, jejunum, ileum and colon of *Bmal1*^{iKO} and control mice (left). mRNA expression of *Bmal1* in liver, kidney, brain, BAT, gWAT and iWAT of *Bmal1*^{iKO} and control mice (right). (**c**) Small intestine weight and length, intestinal motility, villus height, and crypt number of *Bmal1*^{iKO} and control mice. H&E staining of small intestines from *Bmal1*^{iKO} and control mice. Intestinal motility was measured based on whole gut transit assays. 0.15 ml of viscous liquid consisting of 5 % Evans blue (a non-absorbable colored marker) was administered intragastrically to *Bmal1*^{iKO} and control mice. The time was recorded for excretion of the first colored fecal bolus, and was considered as the time of gut transit. (**D**) mRNA expression of clock genes in small intestines of *Bmal1*^{iKO} and control mice. All data points are mean ± SD (n = 6 biologically independent samples). For panels a and c (micrographs), similar results were obtained in three independent experiments.

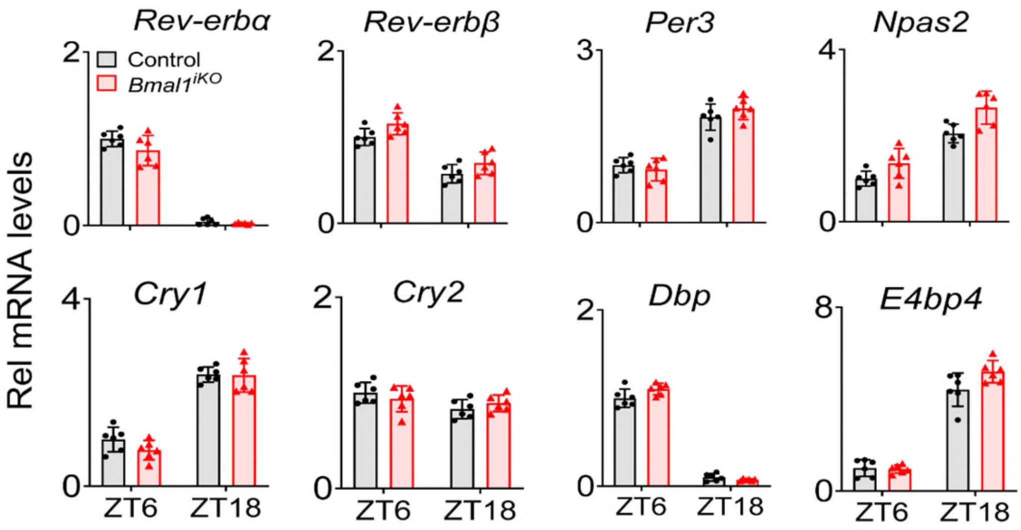

Supplementary Figure 2. mRNA expression of clock genes in livers of *Bmal1*^{iKO} and control mice. Data are mean ± SD (*n* = 6 biologically independent samples).

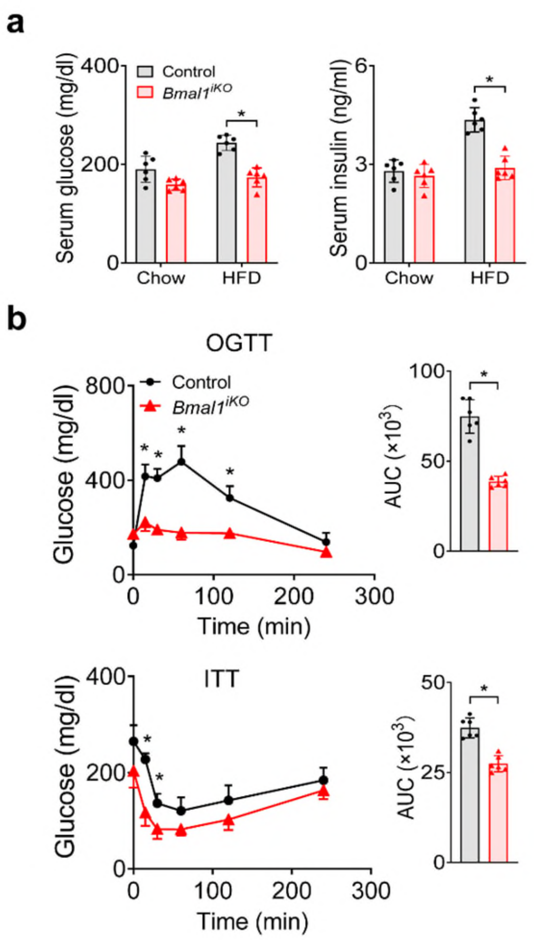

Supplementary Figure 3. (**a**) Fasting serum glucose and insulin levels in *Bmal1*^{iKO} and control mice fed on chow diet or HFD for 10 weeks. Two-sided t test *p* values (from left to right): 0.0514, < 0.0001, 0.4672 and < 0.0001. (**b**) Oral glucose tolerance test (OGTT) and insulin tolerance test (ITT) of *Bmal1*^{iKO} and control mice fed on HFD for 10 weeks. The inserts show the AUC (area under the curve) values for OGTT and ITT. *p* values (OGTT, from left to right): 0.0618, < 0.0001, < 0.0001, < 0.0001, < 0.0001 and 0.0511 (two-way ANOVA and Bonferroni post hoc test). Two-sided t test *p* value: < 0.0001 (OGTT AUC). *p* values (ITT, from left to right): 0.0777, 0.0001, 0.0008, 0.0505, 0.0595 and 0.1389 (two-way ANOVA and Bonferroni post hoc test). Two-sided t test *p* value: < 0.0001 (ITT AUC). Data were shown as mean ± SD (*n* = 6 biologically independent samples). *represents a *p* value of < 0.05.

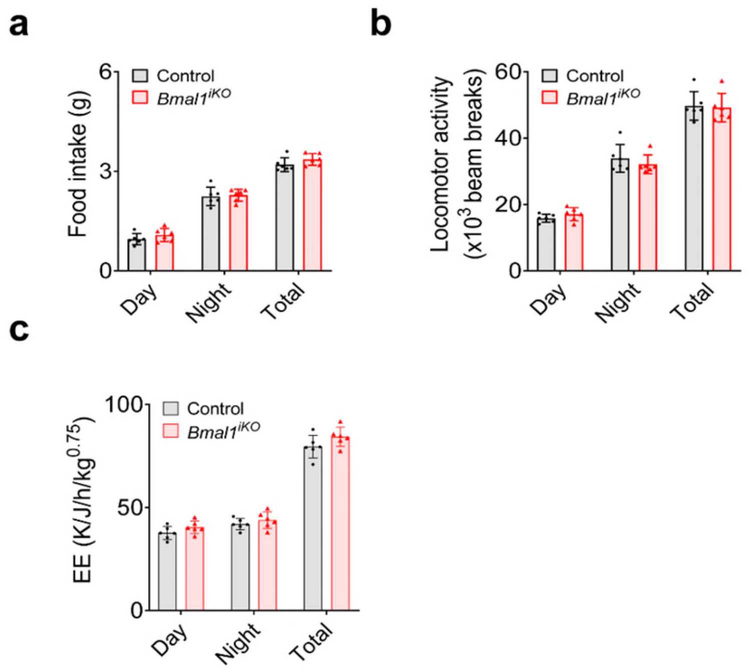

Supplementary Figure 4. (**a**) Daily food intake of *Bmal1*^{iKO} and control mice fed on HFD for 10 weeks. (**b**) Locomotor activities of *Bmal1*^{iKO} and control mice fed on HFD for 10 weeks. (**c**) Energy expenditure (EE) of *Bmal1*^{iKO} and control mice fed on HFD for 10 weeks. Data were shown as mean ± SD (*n* = 6 biologically independent samples).

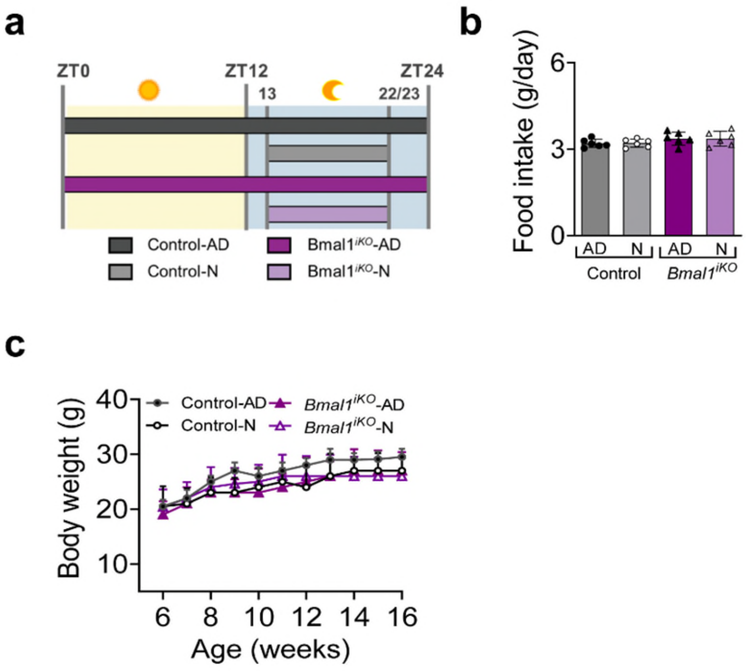

Supplementary Figure 5. (**a**) Schematic diagram of time-restricted (nighttime) feeding and feeding *ad libitum* (on a chow diet). *Bmal1*^{iKO} and control mice with nighttime feeding had access to HFD for 9~10 h in the dark period from ZT13 to ZT22/23. AD, *ad libitum*, N, nighttime. (**b**) Daily food intake of *Bmal1*^{iKO} and control mice fed *ad libitum* or with nighttime feeding. (**c**) Body weight curves of mice over 10 weeks feeding. All data points are mean ± SD (*n* = 6 biologically independent samples).

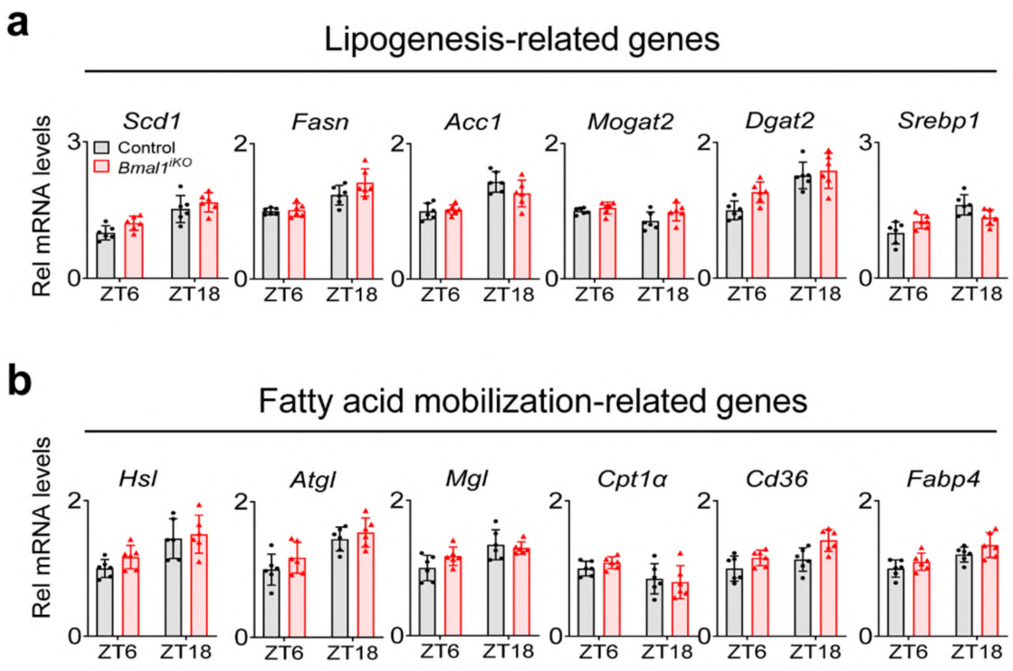

Supplementary Figure 6. (**a**) mRNA expression of lipogenesis-related genes in livers of *Bmal1*^{iKO} and control mice. (**b**) mRNA expression of fatty acid mobilization-related genes in gWAT of *Bmal1*^{iKO} and control mice. Data are mean ± SD (*n* = 6 biologically independent samples).

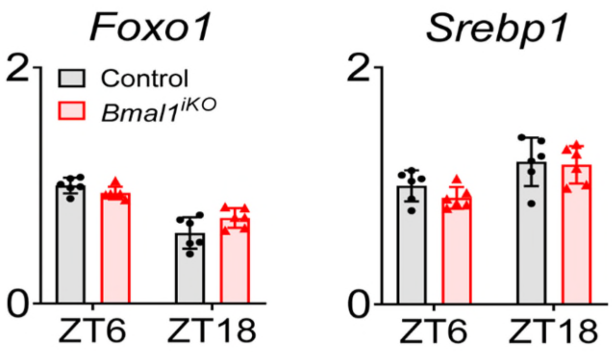

Supplementary Figure 7. mRNA expression of *Foxo1* and *Srebp1* in small intestine of *Bmal1*^{iKO} and control mice at ZT6 and ZT18. Data are mean ± SD (n = 6 biologically independent samples).

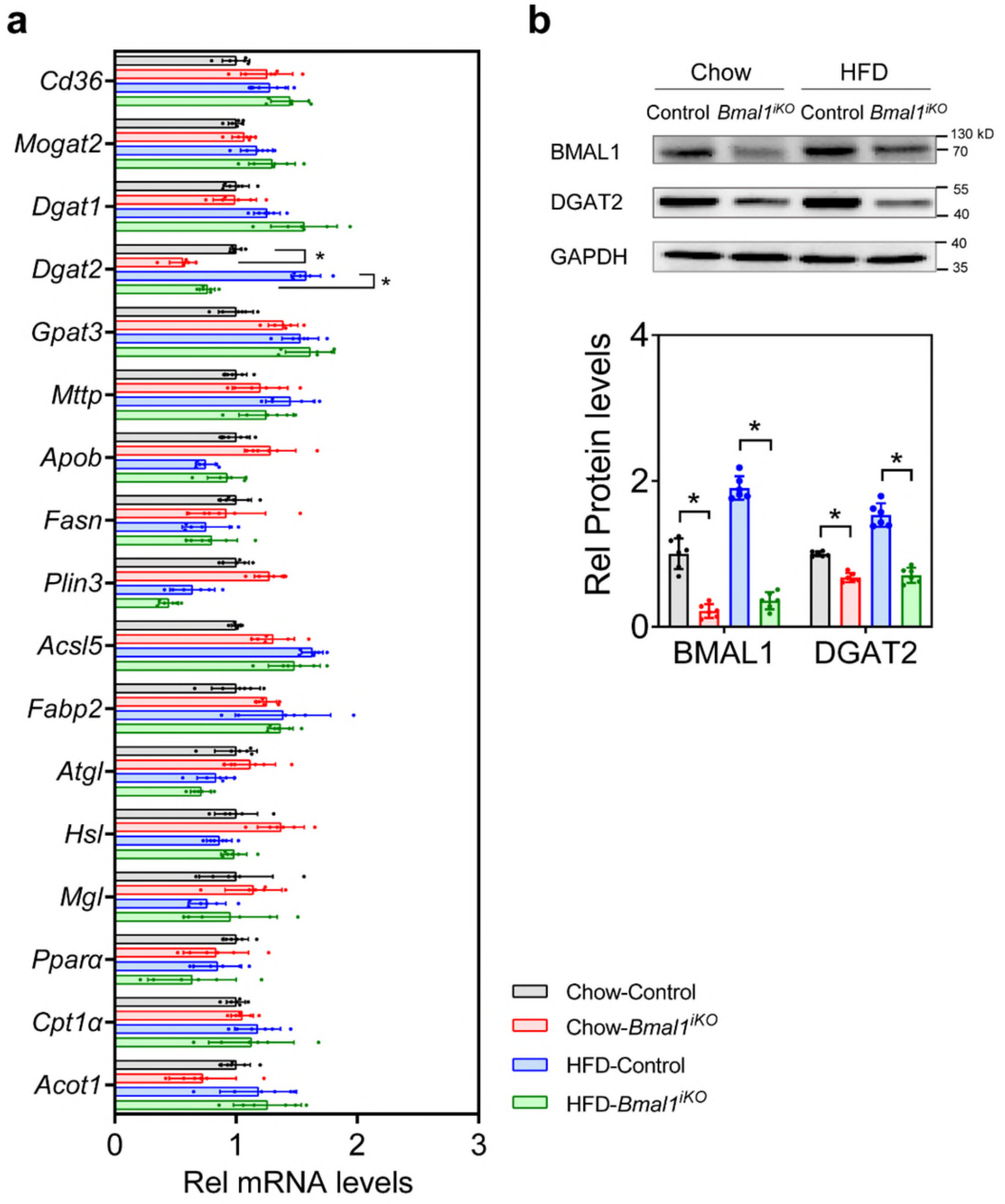

Supplementary Figure 8. mRNA (**a**) and protein (**b**) expression of fat absorption-related genes in small intestine of *Bmal1*^{iKO} and control mice fed a chow diet or HFD. Western blot strips (two target proteins and a loading control) were cut from one gel. Two-sided t test *p* values (panel a, *Dgat2*): < 0.0001 (chow) and $p < 0.0001$ (HFD). Two-sided t test *p* values (panel b, from left to right): < 0.0001, < 0.0001, < 0.0001 and < 0.0001. Data are shown as mean ± SD ($n = 6$ biologically independent samples). For panel b (top), similar results were obtained in six independent experiments. *represents a *p* value of < 0.05.

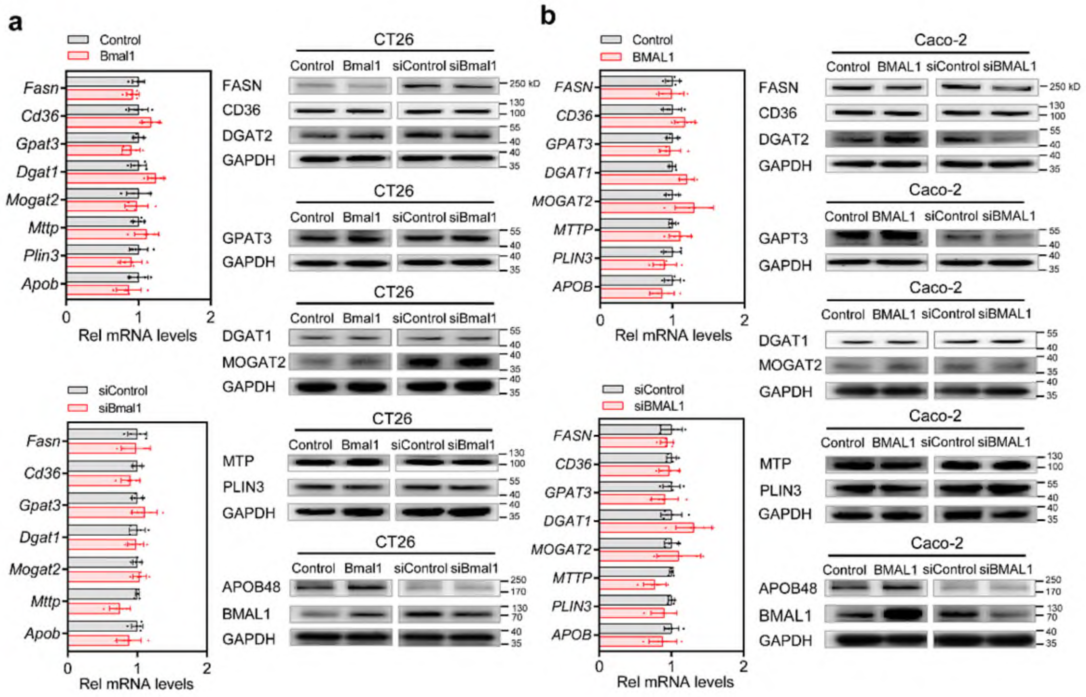

Supplementary Figure 9. (**a**) mRNA and protein expression of fat absorption-related genes in CT26 cells transfected with *Bmal1* plasmid, siBmal1 or control. (**b**) mRNA and protein expression of fat absorption-related genes in Caco-2 cells transfected with *BMAL1* plasmid, siBMAL1 or control. Western blot strips (one or more target proteins and a loading control) were cut from one gel. In particular, the blot of MOGAT2 was stripped and re-probed for GAPDH as a loading control. Data are mean ± SD (*n* = 5 biologically independent samples). For panels a-b (right), similar results were obtained in five independent experiments.

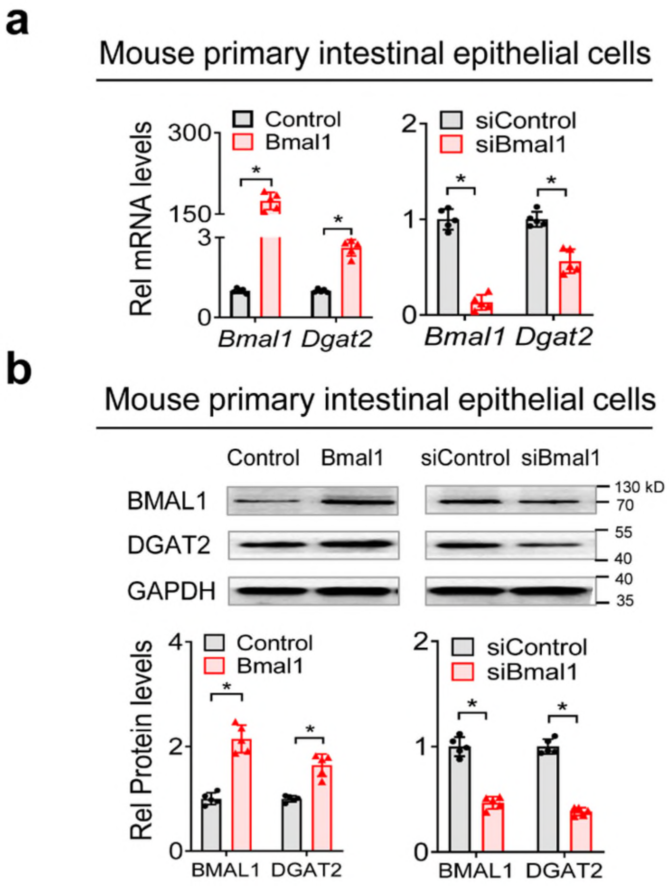

Supplementary Figure 10. (**a**) mRNA expression of *Bmal1* and *Dgat2* in mouse primary intestinal epithelial cells transfected with *Bmal1* plasmid, siBmal1 or control. Two-sided t test *p* values (from left to right): < 0.0001, < 0.0001, < 0.0001 and 0.0002. (**b**) protein expression of BMAL1 and DGAT2 in mouse primary intestinal epithelial cells transfected with *Bmal1* plasmid, siBmal1 or control. Western blot strips (two target proteins and a loading control) were cut from one gel. Two-sided t test *p* values (from left to right): < 0.0001, 0.0002, < 0.0001 and < 0.0001. Data are mean ± SD (*n* = 5 biologically independent samples). For panel b (top), similar results were obtained in five independent experiments. *represents a *p* value of < 0.05.

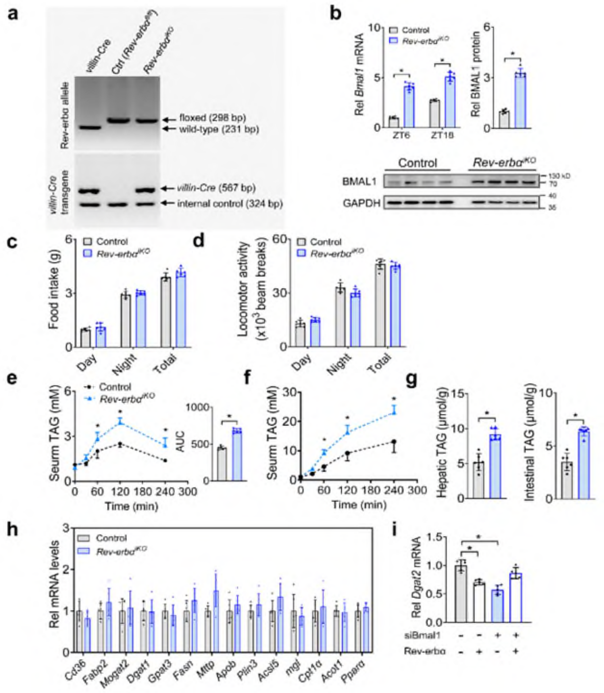

Supplementary Figure 11. (**a**) PCR-based genotyping of *Rev-erbα*^{iKO} mice. *Rev-erbα*^{iKO} mice had both floxed *Rev-erbα* allele and *villin-Cre*. (**b**) mRNA (*n* = 6 biologically independent samples) and protein (*n* = 4 biologically independent samples) expression of BMAL1 in small intestines of *Rev-erbα*^{iKO} and control mice. Western blot strips (a target protein and a loading control) were cut from one gel. Two-sided t test *p* values (from left to right): 0.0001, 0.0002 and < 0.0001. (**c**) Daily food intake of *Rev-erbα*^{iKO} and control mice fed on HFD for 10 weeks. Data are mean ± SD (*n* = 6 biologically independent samples). (**d**) Locomotor activities of *Rev-erbα*^{iKO} and control mice fed on HFD for 10 weeks. Data are mean ± SD (*n* = 6 biologically independent samples). (**e**) Serum TAG levels in *Rev-erbα*^{iKO} and control mice after oral gavage of olive oil (10 μl/g). The insert shows the AUC values for serum TAG. *p* values (from left to right): 0.3328, 0.0835, 0.0451, < 0.0001 and 0.0311 (two-way ANOVA and Bonferroni post hoc test). Two-sided t test *p* value (AUC): <0.0001. Data are mean ± SD (*n* = 6 biologically independent samples). (**f**) Serum TAG levels in tyloxapol (500 mg/kg, i.p., 30 min)-pretreated *Rev-erbα*^{iKO} and control mice after olive oil gavage. *p* values (from left to right): 0.2177, 0.0681, 0.0004, 0.0005 and 0.0037 (two-way ANOVA and Bonferroni post hoc test). Data are mean ± SD (*n* = 6 biologically independent samples). (**g**) Hepatic and intestinal TAG levels in *Rev-erbα*^{iKO} and control mice at 2 h after oil gavage. Two-sided t test *p* values: 0.0468 and 0.0481. Data are mean ± SD (*n* = 6 biologically independent samples). (**h**) mRNA expression of fat absorption-related genes in small intestines of *Rev-erbα*^{iKO} and control mice. Data are mean ± SD (*n* = 6 biologically independent samples). (**i**) Knockdown of *Bmal1* attenuates the inhibition effects of *Rev-erbα* on *Dgat2* expression. CT26 cells were transfected with indicated plasmids. After 48 h, mRNA was extracted and quantified by qPCR. Data are mean ± SD (*n* = 5 biologically independent samples). Two-sided t test *p* values (from left to right): 0.0003, 0.0001 and 0.0603. For panels a and b (bottom), similar results were obtained in four independent experiments.*represents a *p* value of < 0.05.

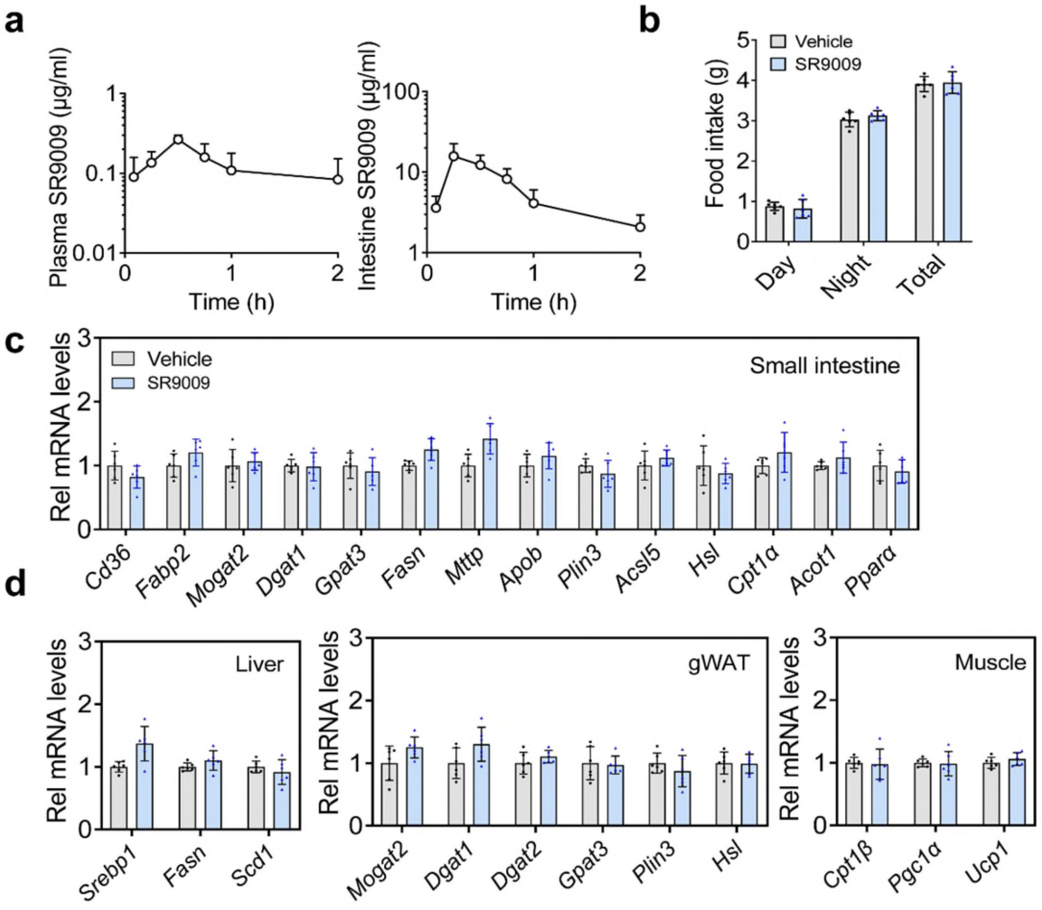

Supplementary Figure 12. (**a**) Plasma concentration-time and intestine concentration-time profiles of SR9009 after oral gavage (100 mg/kg). (**b**) Daily food intake of SR9009- and vehicle-treated mice. (**c**) mRNA expression of fat absorption-related genes in small intestine of SR9009-treated mice. (**d**) mRNA expression of lipogenic genes in the liver, TAG synthesis genes in gWAT, and genes involved in fatty acid oxidation in skeletal muscle of SR9009-treated mice. Data are shown as mean ± SD (n = 6 biologically independent samples).

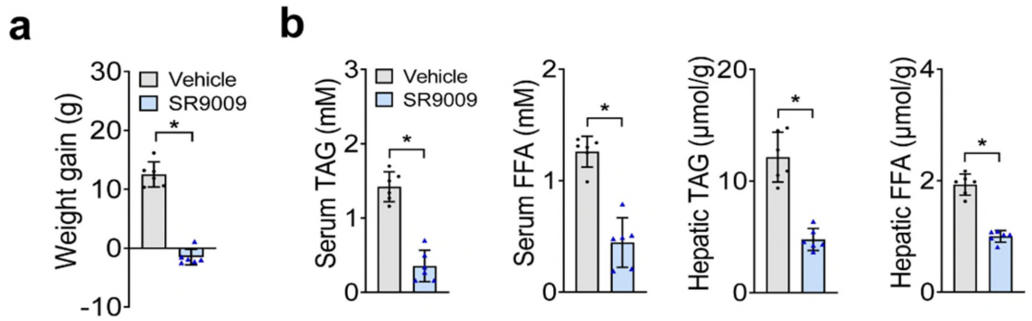

Supplementary Figure 13. (**a**) Effects of oral SR9009 on body weight gain in HFD-fed mice. Mouse treatment: 6-week-old mice had been kept on chow diet. The mice were switched to HFD feeding and gavaged with SR9009 (100 mg/kg) or vehicle at ZT2 once daily for 8 weeks and body weights were measured. Two-sided t test *p* value: 0.0001. (**b**) Serum and hepatic TAG and FFA levels in SR9009-treated mice. Two-sided t test *p* values (from left to right): 0.0001, 0.0002, 0.0002 and < 0.0001. Data are mean ± SD (*n* = 6 biologically independent samples). *represents a *p* value of < 0.05.

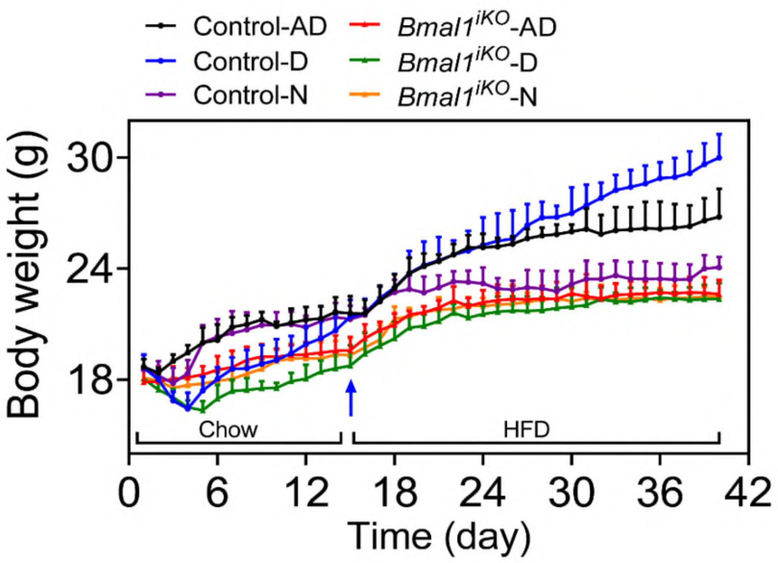

Supplementary Figure 14. Body weight curves of *Bmal1*^{iKO} and control mice fed the same amount of daily calories with access to food only during daytime (D), only during nighttime (N) or *ad libitum* (AD). Mice required an adjustment period to the feeding regimen, after which they were introduced to HFD (day 16). Data are mean ± SD (*n* = 8 biologically independent samples).

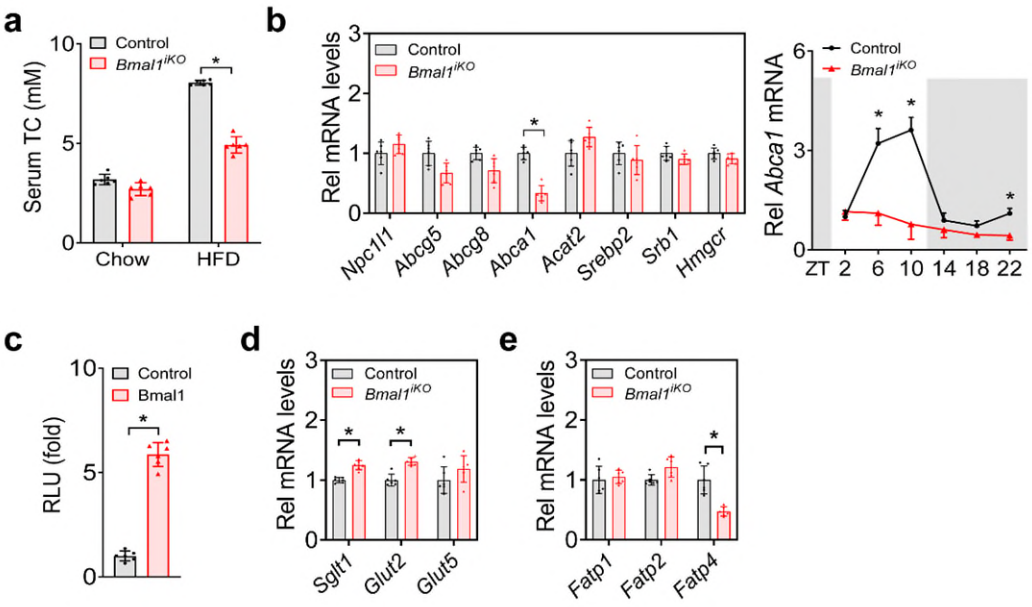

Supplementary Figure 15. (**a**) Serum total cholesterol (TC) levels in *Bmal1*^{iKO} and control mice fed on chow or HFD for 10 weeks. Two-sided t test *p* values: 0.0508 (chow) and < 0.0001 (HFD). (**b**) mRNA expression of cholesterol metabolism-related genes in small intestines of *Bmal1*^{iKO} and control mice (left). Diurnal *Abca1* mRNA expression in small intestine of *Bmal1*^{iKO} and control mice (right). Two-sided t test *p* value in left panel (*Abca1*): < 0.0001. *p* values in right panel (from left to right): 0.1906, < 0.0001, < 0.0001, 0.0631, 0.0501 and < 0.0001 (two-way ANOVA and Bonferroni post hoc test). (**c**) *Bmal1* induces *Abca1* transcription in luciferase reporter assay. NIH3T3 cells were transfected with *Abca1*-Luc reporter and *Bmal1* plasmid. After 24 h, luciferase reporter activities were measured. Two-sided t test *p* value: < 0.0001. (**d**) mRNA expression of carbohydrate transporters in small intestines of *Bmal1*^{iKO} and control mice. Two-sided t test *p* values (from left to right): < 0.0001, 0.0050 and 0.2147. (**e**) mRNA expression of fatty acid uptake genes in small intestines of *Bmal1*^{iKO} and control mice. Two-sided t test *p* values (from left to right): 0.0945, 0.0657 and 0.0004. Data are mean ± SD (*n* = 6 biologically independent samples). *represents a *p* value of < 0.05.
